# Musical Melody and Speech Intonation: Singing a Different Tune

**DOI:** 10.1371/journal.pbio.1001372

**Published:** 2012-07-31

**Authors:** Robert J. Zatorre, Shari R. Baum

**Affiliations:** 1 Montreal Neurological Institute, McGill University, Montreal, Quebec, Canada; 2 Centre for Research on Brain, Language & Music, McGill University, Montreal, Quebec, Canada; 3 School of Communication Sciences and Disorders, McGill University, Montreal, Quebec, Canada

## Abstract

Music and speech are often cited as characteristically human forms of communication. Both share the features of hierarchical structure, complex sound systems, and sensorimotor sequencing demands, and both are used to convey and influence emotions, among other functions [1]. Both music and speech also prominently use acoustical frequency modulations, perceived as variations in pitch, as part of their communicative repertoire. Given these similarities, and the fact that pitch perception and production involve the same peripheral transduction system (cochlea) and the same production mechanism (vocal tract), it might be natural to assume that pitch processing in speech and music would also depend on the same underlying cognitive and neural mechanisms. In this essay we argue that the processing of pitch information differs significantly for speech and music; specifically, we suggest that there are two pitch-related processing systems, one for more coarse-grained, approximate analysis and one for more fine-grained accurate representation, and that the latter is unique to music. More broadly, this dissociation offers clues about the interface between sensory and motor systems, and highlights the idea that multiple processing streams are a ubiquitous feature of neuro-cognitive architectures.

## Abstract 2

Pitch changes are an integral part of both spoken language and song. Despite sharing some of the same psychological and neural mechanisms, the authors conclude there are fundamental differences between them.

Whether you speak or sing, your vocal tract modulates the pitch of your voice. But to what extent do the mechanisms for producing and perceiving pitch in speech differ from those enlisted in musical contexts? Here we discuss the relevant evidence from psychology and neuroscience. We propose that although speaking and singing involve a substantial sharing of resources, musical pitch requires more accurate encoding and reproduction of pitch relationships than does speech.

## Similarities in the Use of Pitch in Music and Speech

The importance of pitch for melodic processing needs little justification; it is hard to imagine a musical system that does not include more than a single pitch (Antonio Carlos Jobim's “One-Note Samba” notwithstanding). Things are more complicated in the case of speech, where pitch variation forms part of a more complex set of modulations known as prosody. Prosody refers to the set of speech parameters that generally apply across individual speech sounds (i.e., at the level of the syllable, phrase, or sentence), including intonation (fundamental frequency, corresponding to pitch variations across a sentence), stress, and rhythm. Prosody is particularly useful in various communicative functions of language, including distinguishing word meanings in tone languages (e.g., Mandarin and Thai), disambiguating sentence structures (e.g., distinguishing questions from statements), highlighting or emphasizing elements in a sentence, and signaling emotion (including irony and sarcasm). Whereas all of the prosodic parameters contribute in varying ways to these functions, for the purpose of the present discussion, we will concentrate on the most evident parallel in music and speech—the processing of melody and sentence-level intonation, or pitch.

**Figure 1:**
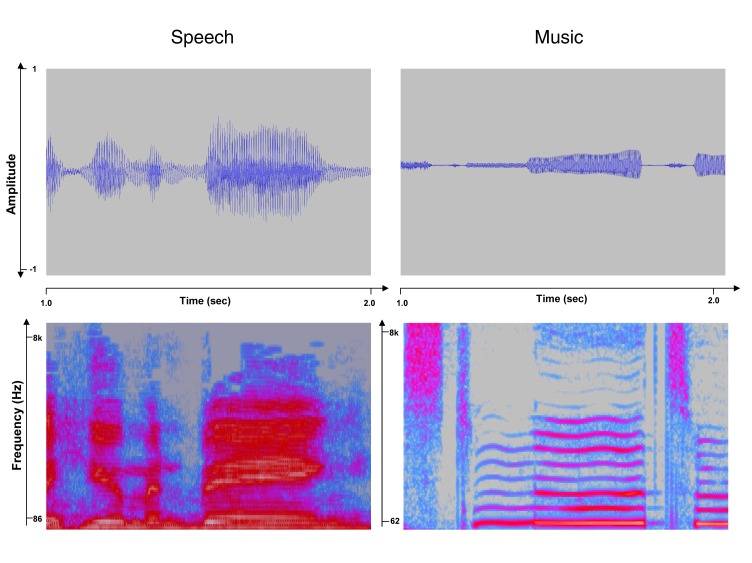
Acoustical representations of speech and song. The top panels show the waveforms (amplitude as a function of time) of 2-s excerpts of samples of spoken and sung speech, respectively. The bottom panels show spectrograms (frequency as a function of time) of the same sound samples; intensity is coded by a color scale in this representation. Note the prominent fundamental frequency and harmonics (horizontal lines) present in the sung speech.

Both speech and music production rely on the ability to control the tension on the vocal cords, which (in combination with transglottal air pressure) results in modulations of the vocal fundamental frequency (Figure 1). Recent acoustical analyses suggest that the probability distribution of the amplitudes of harmonics present in human speech can be used to predict the structure of musical scales, in terms of the pitch intervals that are most commonly used across cultures [2]. These data can also lead to predictions about consonance judgments of pitches drawn from these scales [3]. There may therefore be a close connection between vocalizations and the tonal structure of musical scales, at least in terms of origins, which in turn implies a close connection between production and perception of both music and speech.

## Differences in the Use of Pitch between Music and Speech

**Figure 2:**
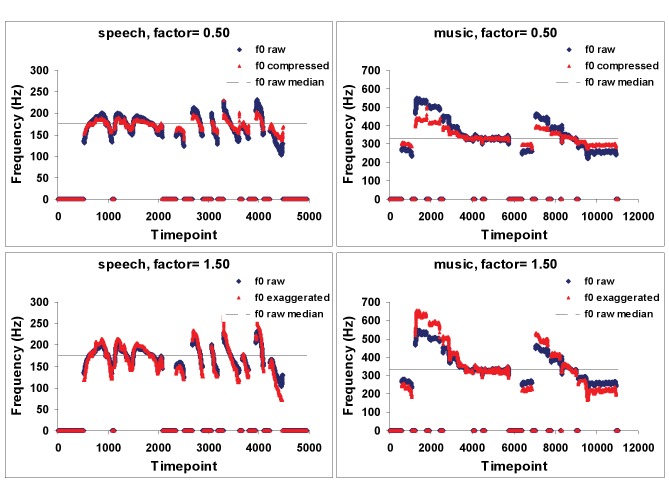
Each panel represents the fundamental frequency (F0) contour of a spoken utterance (left side of figure) or of sung speech (right side of figure). Note the more continuous F0 contours for speech compared to the more discrete contours for song. The blue traces are the original contours, while the red ones represent distortions in which the F0 was either compressed by 50% (top panels) or exaggerated by 50% (bottom panels). The associated sound files illustrate that the manipulation of F0 on the speech sample (Sounds S1, S2, S3) has little perceptual effect, since it continues to sound natural (in fact, the change is hardly detectable). In contrast, the same degree of F0 distortion on the music (Sounds S4, S5, S6) is readily noticeable, as the familiar melody sounds obviously out of tune.

Despite these fundamental similarities between the use of pitch in speech and in music, closer inspection reveals some critical differences between the two domains. Although under some unusual conditions spoken speech may be perceived as sung [4], the two are rarely confused. One reason that song and speech are clearly different is that pitch variations in melodies are mostly discrete, compared to those in speech, which are continuous (Figure 2). Music from a wide array of different cultures throughout the world most often uses pitches drawn from a limited set of tones (commonly five or seven) within an octave, creating scales that have specific musical interval values [2]; there is no counterpart of this phenomenon in speech intonation. Furthermore, the various tones within a scale are hierarchically organized and play different roles in most musical systems, leading to a wide array of perceptual phenomena (such as key structures, harmonic relationships, etc.) that may be subsumed under the term tonality [5]; again, there is no truly analogous feature in speech intonation.

Most importantly, pitch within music depends on a much greater degree of accuracy, both in production and perception, as compared to speech. Many musical systems, including the Western tonal one, depend on specific, fixed musical intervals (frequency ratios). Under most circumstances, even fairly small deviations from these prescribed intervals are readily perceived as errors by listeners [6]. In contrast, only rough frequency relationships are important for speech intonation: deviations of a similar magnitude as those that sound wrong in a melody are not perceived as violations in a speech contour. Behavioral studies show that removing all fundamental frequency modulation does not affect speech comprehension, even for tonal languages [7] unless the content is ambiguous [8] or the signal-to-noise ratio is poor [9]. The sound examples (Figure 2) illustrate that accurate pitch relationships are more important for music than for speech: compare a 50% change in the magnitude of the pitch intervals (expansion or contraction) applied to a natural speech sample with the identical manipulation applied to a song. The speech sounds fairly natural under all conditions, whereas the song is clearly out of tune when the pitch is altered; indeed, the concept of “out of tune” does not even really apply to speech. Thus, there is a profound difference in how pitch is used in speech and music.

## Fine Versus Coarse Pitch Representations

**Figure 3:**
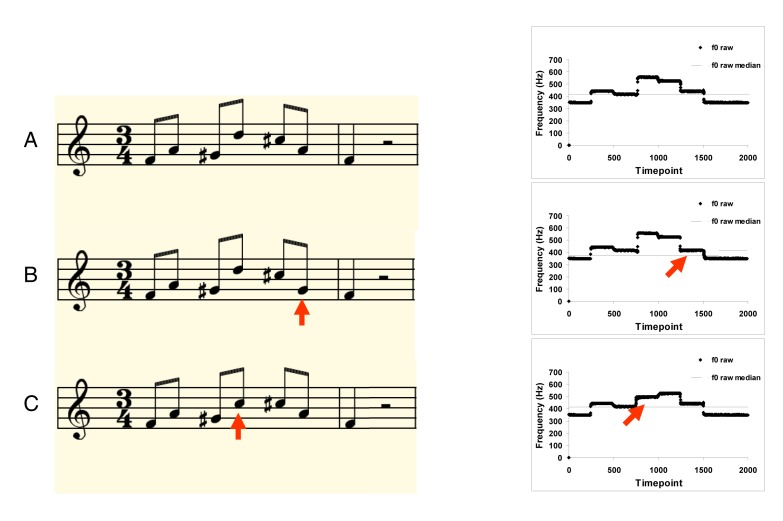
Three melodies in musical notation (left) and their corresponding fundamental frequency contours (right). Melodies B and C are identical to Melody A, except for one changed tone (indicated by red arrows in both the musical notation and the pitch traces). Melodies A and B have the same contour (up, down, up, down, down, down), whereas Melody C has a different contour (up, down, up, up, down, down). The associated sound files illustrate that Melody C (Sound S9) is generally more easily distinguished from Melody A (Sound S7) because of this contour change, whereas Melody B (Sound S8) sounds more similar to Melody A because it has the same contour.

One way to think about the different uses of pitch variation in music and speech is to distinguish between the fine-grained, accurate encoding required for processing musical interval relationships used in scales, as compared to the more coarse-grained processing associated with contours. Contour in both music and speech is defined by the direction of pitch changes, but not by specific pitch relationships. Contour is especially relevant for speech, since direction of intonation can change linguistic meaning (e.g., question versus statement, or rising versus falling tones in Mandarin). But contour also plays a fundamental role in music perception: cognitive studies have shown that contour information is more perceptually salient (Figure 3) and more easily remembered, whereas specific intervals take more time to encode [10]. Infants detect contour but not interval information [11], implying that it is a more basic process that develops early or is innate. The neural correlates of contour and scale processing also appear to differ [12],[13]. Taken together, these findings suggest that perhaps the coarse pitch processing related to contour might represent one mechanism used for both speech and music, whereas the precise encoding and production required for musical scale information might be a separate mechanism, perhaps even one that emerged later in phylogeny.

## Dual Processing in the Brain for Music Versus Speech

Consistent with this proposal, there is a large amount of human lesion evidence indicating that the processing of speech prosody and the processing of melody in music may be partially dissociated. Numerous investigations of individuals who have suffered focal brain damage (particularly within the right cerebral hemisphere) have demonstrated impairments in the ability to convey and/or perceive or comprehend speech intonation and its functional significance [14],[15]. In fact, although lesions in the left hemisphere (LH) have long been associated with impaired comprehension of linguistic meanings conveyed by prosody, rarely have isolated LH lesions been reported to lead to major disorders of melody perception [16],[17]. In contrast, however, evidence also exists supporting the notion of a shared neural substrate for the processing of melody in speech and music. For example, there have been a number of studies of patients with documented lesions that result in music processing deficits that have reported parallel difficulties in the perception of speech prosody [18],[19]. Such patterns of partially shared but dissociable processing mechanisms fit well with our hypothesis of dual processing mechanisms for pitch perception.

Functional imaging studies show evidence both for segregation and overlap in the recruitment of cortical circuits for perception of speech and of tonal patterns [20]–[23], but the commonalities may be more apparent than real. Sharing is likely due either to common task demands (for example, working memory) or to common input or output systems, with distinct neural resources at other levels [24],[25]. Moreover, there is consistent evidence for a relative advantage of right auditory cortical structures compared to left for fine-grained spectral processing [26]–[28]. Similarly, when contrasting vocal pitch production in linguistic and musical contexts, there seems to be overlap, but greater reliance on right-hemisphere structures during singing compared to speaking [29]. Imaging studies of trained singers [30],[31] indicate that singing involves specialized contributions of auditory cortical regions, along with somatosensory and motor-related structures, suggesting that singing makes particular demands on auditory-vocal integration mechanisms related to the high level of pitch accuracy required for singing in tune, which is less relevant for speech.

The distinction between two pitch mechanisms finds additional support from amusia, because a dissociation can be seen between preserved contour but impaired fine-pitch processing. People with congenital amusia, also known as tone-deafness, have little difficulty perceiving large changes in pitch contours typical of speech [32]. When measured with stimuli that have small pitch deviations, however, these individuals show impairments, whether the stimuli are speech or not [33],[34], indicating a selective deficit at the level of fine-grained pitch distinctions [35], which are not as critical for speech as they are for music, as we have seen. These behavioral data fit with evidence of anatomical [36],[37] and functional [38] disruption in right auditory-frontal cortical circuitry, consistent with the functional neuroimaging evidence cited above suggesting that this circuitry plays a role in fine-grained pitch processing.

## Potential Subcortical Mechanisms for Processing Music and Speech

If pitch processing for speech and music are dissociable at the cortical level, it is fair to ask if the dissociation originates there or at subcortical levels. Auditory brainstem activity can be studied using an electrical evoked potential measure, the frequency-following response, which most likely originates in the inferior colliculus. As its name implies, it encodes the frequency information contained in the acoustic stimulus in terms of changes in voltage that follow the fundamental frequency of the stimulus. Several studies have shown that the fidelity of the brainstem response in relation to the frequency content of the stimulus is enhanced both in tone-language speakers [39] and in trained musicians [40]. Moreover, training in one domain results in generalization of the brainstem enhancement in the other domain, such that musicians show better encoding of linguistic tone while tone-language speakers show enhancement for musical tones [41],[42]. This reciprocity suggests that the distinctions seen at cortical levels have not yet emerged at the subcortical processing stage. Yet the origins of this experience-dependent modulation are not fully understood. Differences as a function of training in very early latencies of brainstem onset responses, before activity in auditory cortex [40], suggest that part of the enhancement is intrinsic to the brainstem. However, it could also be the case that cortical efferent mechanisms are also at play in the frequency following response.

## Conclusion

In summary, the evidence indicates that despite some shared cognitive processes and neural substrates, the way pitch information is handled in speech and in music differs: there seem to be two mechanisms, one focused on contour, which may overlap across domains, and another, perhaps specific to music, involving more accurate pitch encoding and production. This distinction is reminiscent of parallel processing in other neural domains, such as vision, memory, or the motor system, where multiple types of analysis are needed to solve distinct problems. The dissociation we have discussed for pitch may therefore be seen as one more example of this more general biological principle.

One implication of this model is that it should be possible to identify distinct neural substrates for the two mechanisms. Although some of the evidence points in this direction, there is no firm identification of the underlying neural circuitry that may give rise to the two processes. How the two hypothesized mechanisms emerge from interactions between cortical and subcortical pitch-processing mechanisms also remains to be understood. It might also be valuable to consider the distinction we have drawn in evaluating comparative analyses of how different animal species make use of pitch for communicative purposes [43],[44]. A greater understanding of the neural circuitry involved in the perception and production of pitch across cognitive domains will permit us to develop a more advanced model of the sensorimotor control of communicative systems, from basic processing to integration with higher order linguistic and cognitive processes beyond auditory and motor cortices [45],[46]. We believe that substantial advances will emerge from such interdisciplinary ventures, with potential for future applications in fields as diverse as computer voice recognition to the rehabilitation of individuals who have suffered brain damage.

## Supporting Information

Sound S1 Original speech.
(WAV)

Sound S2 Pitch-compressed speech.
(WAV)

Sound S3 Pitch-expanded speech.
(WAV)

Sound S4 Original song.
(WAV)

Sound S5 Pitch-compressed song.
(WAV)

Sound S6 Pitch-expanded song.
(WAV)

Sound S7 Melody A.
(WAV)

Sound S8 Melody B.
(WAV)

Sound S9 Melody C.
(WAV)
